# Thermal runaway-free Na-ion batteries

**DOI:** 10.1093/nsr/nwag279

**Published:** 2026-05-13

**Authors:** Runtian Zheng, Jie Shu, Yu Li, Bao-Lian Su

**Affiliations:** Laboratory of Inorganic Materials Chemistry (CMI), University of Namur, Belgium; School of Chemistry and Chemical Engineering, Shaoxing University, China; State Key Laboratory of Advanced Technology for Materials Synthesis and Processing, Wuhan University of Technology, China; Laboratory of Inorganic Materials Chemistry (CMI), University of Namur, Belgium; State Key Laboratory of Advanced Technology for Materials Synthesis and Processing, Wuhan University of Technology, China

Na-ion batteries (NIBs) have emerged as promising candidates for large-scale energy storage owing to their abundant resources, low cost, and favorable low-temperature performance [1–3]. However, similar to Li-ion batteries, they typically rely on organic electrolytes that are highly flammable, posing serious safety risks such as thermal runaway under extreme conditions [4–6]. Therefore, the development of high-energy-density and non-flammable electrolyte systems is critical to achieving safe and practical NIBs. Conventional strategies have largely focused on developing non-flammable electrolytes, under the assumption that suppressing flammability would directly translate into enhanced battery safety. However, electrolytes with superior flame retardancy, such as phosphate-based systems, could still fail to prevent catastrophic thermal events under extreme conditions [7].

**Figure 1.**
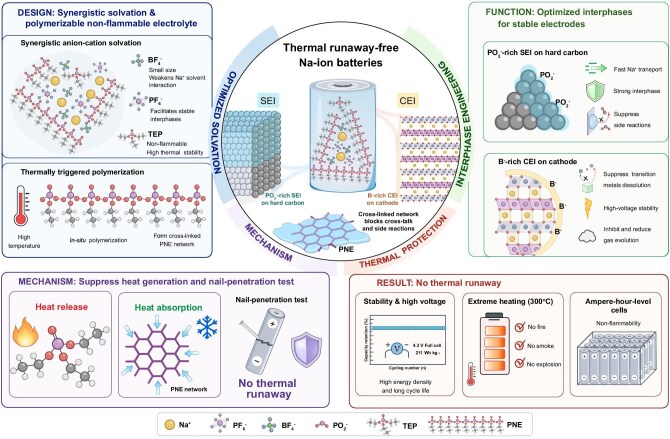
Illustration of the polymerizable non-flammable electrolyte enabling thermal runaway-free NIBs. SEI: solid electrolyte interface; CEI: cathode electrolyte interface.

In a recent study published in *Nature Energy*, entitled ‘Thermal runaway-free ampere-hour-level Na-ion battery via polymerizable non-flammable electrolyte’, Hu and co-workers report an ampere-hour-level sodium-ion battery that is free of thermal runaway, enabled by a polymerizable non-flammable electrolyte, as illustrated in Fig. 1 [8]. From a design perspective, the electrolyte integrates triethyl phosphate (TEP) as a non-flammable solvent and employs a dual-anion strategy (BF_4_^−^/PF_6_^−^) to regulate the Na^+^ solvation structure. This synergistic solvation environment weakens cation–solvent interactions and promotes the formation of stable electrode/electrolyte interphases. More importantly, the electrolyte exhibits a unique function with thermally triggered *in-situ* polymerization behavior. Upon heating, TEP-derived species undergo polymerization to form a cross-linked non-flammable electrolyte (PolyNonflyte or PNE), which acts as an insulating network that physically separates the electrodes and suppresses interfacial reactions. As a result, this multifunctional electrolyte enables unprecedented safety performance at the cell level. Ampere-hour-scale cylindrical NIBs (up to 3.5 Ah) demonstrate complete elimination of thermal runaway, even under extreme conditions such as nail penetration and heating up to 300°C, without smoke, fire, or explosion.

Unlike traditional carbonate electrolytes, which are highly flammable and prone to exothermic decomposition, or phosphate-based electrolytes that are non-flammable yet still thermally unstable at the cell level, the PNE system provides an additional layer of protection through thermal responsiveness. Compared with high-concentration or fluorinated electrolytes that rely on modifying solvation structures at the expense of cost and complexity, PNE achieves both interfacial stability and safety without excessive salt loading or fluorination. Furthermore, the PNE actively transforms into a protective polymer barrier, thereby suppressing electrode cross-talk, parasitic reactions, and reductive gas evolution. This adaptive behavior represents a significant advancement over static electrolyte designs and offers a more robust route toward safe batteries.

Mechanistically, the superior safety performance originates from a synergistic interplay between solvation chemistry, interphase engineering, and thermally induced structural evolution. The dual-anion system of BF_4_^−^/PF_6_^−^ tailors the primary solvation sheath, facilitating Na^+^ desolvation and enabling the formation of PO_2_^−^-rich solid electrolyte interphase and B^−^-rich cathode electrolyte interphase layers. These interphases enhance electrochemical stability while suppressing transition metal dissolution and side reactions. Upon thermal abuse, the electrolyte undergoes endothermic decomposition coupled with polymerization, which not only absorbs heat but also generates a cross-linked network that immobilizes reactive species and prevents electrode contact. This mechanism effectively interrupts the chain reactions responsible for thermal runaway. Looking forward, this research suggests a new design principle for battery safety, which combines passive stability with active stimulus response protection. Such strategies could be extended to other battery chemistries, including Li-ion batteries and other systems, and may inspire the development of next-generation ‘smart electrolytes’.

Overall, this work demonstrates a transformative electrolyte design that enables thermal runaway-free operation in ampere-hour-level cells. By combining nonflammability, interfacial optimization, and thermally triggered polymerization, the PNE system transcends conventional safety paradigms and establishes a new benchmark for battery safety. Beyond its immediate implications for NIBs, this strategy offers a broadly applicable framework for designing safer, high-performance energy storage systems. As the demand for grid-scale storage continues to grow, such intrinsically safe and scalable solutions will be crucial for accelerating the widespread adoption of electrochemical energy technologies.
